# Clinical Study on the Application of Acupuncture in the Postoperative Rehabilitation of Dogs Affected by Acute Thoracolumbar Disc Herniation

**DOI:** 10.3390/ani15081154

**Published:** 2025-04-17

**Authors:** Michela Antonucci, Erika Passarini, Enrico Bruno, Thomas Dalmonte, Giuseppe Spinella

**Affiliations:** 1 Ospedale Veterinario I Portoni Rossi-Anicura, Via Roma, 57/A, 40069 Zola Predosa, BO, Italyenrico.bruno@anicura.it (E.B.); 2 Department of Veterinary Medical Sciences, Alma Mater Studiorum—University of Bologna, Via Tolara di Sopra 50, 40064 Ozzano dell’Emilia, BO, Italy; thomas.dalmonte2@unibo.it

**Keywords:** acupuncture, electro-acupuncture, acute disc herniation, rehabilitation, dogs

## Abstract

Acupuncture has been widely incorporated into rehabilitation protocols for dogs and cats because of its potential analgesic efficacy. The aim of this study was to evaluate the potential positive effect of integrating acupuncture and electroacupuncture techniques on the recovery of ambulation capacity in non-ambulating paraparetic patients undergoing physiotherapy in the postoperative period following mini-/hemilaminectomy for thoracolumbar spinal cord decompression due to acute disc extrusion. Forty-one patients were included and underwent descriptive and analytical statistical analysis, divided into two groups: dogs that received a physiotherapy protocol with acupuncture and dogs that received physiotherapy only. The results showed that the dogs in the acupuncture group had a higher likelihood of regaining ambulation.

## Simple Summary

This study investigated the efficacy of acupuncture and electroacupuncture within a physiotherapy protocol for the postoperative rehabilitation of dogs affected by acute disc extrusion, as a multimodal approach for faster recovery of ambulation. Results from 41 patients indicated that the inclusion of acupuncture in the rehabilitation protocol led to a higher probability of regaining the ability to ambulate.

## 1. Introduction

Acupuncture is a widely practiced therapeutic technique that involves the insertion of fine needles into specific points on the body to stimulate the flow of vital energy (‘Qi’) and promote the restoration of physical and mental balance in the organism [1]. According to traditional Chinese medicine [2], health results from the harmonious circulation of Qi throughout the body along specific pathways known as ‘channels’ or ‘meridians’. Acupuncture treatment is tailored to the patient’s needs, aiming to address the origin of the disorder and prevent recurrences by rebalancing energy [1,3].

Scientific research on acupuncture as a medical treatment for humans has grown exponentially over the past 20 years. Most studies have been focused on pain management [4,5], as well as on other pathological conditions related to neurological disorders such as paresis or paralysis, gastrointestinal diseases, allergies, and immune system disorders [1,3,6,7]. Additionally, acupuncture has been associated with conditions such as epilepsy, hormonal imbalances, dermatological and behavioral disorders, and vomiting and nausea [1,3]. However, the scientific community still requires further research to validate the clinical outcomes achieved so far, both in human and veterinary medicine.

Intervertebral disc disease (IVDD) is a neurological condition resulting from the protrusion or extrusion of the nucleus pulposus into the spinal canal, which can lead to compressive myelopathy [8]. Excluding traumatic forms, IVDD is a pathological condition caused by intervertebral disc degeneration, which may result in intervertebral disc herniation, involving the protrusion or extrusion of the disc or parts of it from its usual position [9].

Hansen Type I intervertebral disc extrusion, or Type I disc herniation, is a very common cause of acute paraparesis and paraplegia in dogs. This injury predominantly affects chondrodystrophic canine breeds, with the highest incidence observed in dogs aged 3 to 6 years. The predisposition is particularly linked to the expression of a fibroblast growth factor 4 (FGF4) retrogene on chromosome 12, which is associated with dramatically accelerated intervertebral disc degeneration [8,10,11].

In thoracolumbar extrusions, symptom severity can be classified using a grading scale that evaluates focal hyperesthesia as well as motor, sensory, and urinary function [10]. Clinical signs include kyphosis, proprioceptive ataxia, paraparesis, paraplegia with or without loss of deep nociception, and other neurological deficits [12]. A consistent feature of disc extrusions is the acute onset of spontaneous pain, which may not always be accompanied by clear neurological symptoms. In less severe cases, animals may show only reluctance to move.

Neurological rehabilitation with a suitable exercise protocol may have the ability to modulate neuroinflammation and stimulate neurotrophins; moreover, these effects could be enhanced if physical therapy is associated with specific modalities such as neuromuscular electrical stimulation (NMES), photobiomodulation (laser therapy), and acupuncture [13]. According to the veterinary scientific literature, few studies have investigated the use of acupuncture techniques within a structured physiotherapy protocol for the post-surgical treatment of neurological conditions [14,15,16,17]. Acupuncture may promote analgesia, stimulate trophic factors, decrease inflammation, and reduce muscular atrophy [13]. Moreover, as observed by Santos et al., it may increase conscious proprioception [18].

The aim of this study was to evaluate the potential positive effect of integrating acupuncture and electroacupuncture (EA) techniques on the recovery of ambulation in non-ambulating patients undergoing physiotherapy during the postoperative period following mini-/hemilaminectomy for thoracolumbar (T3–L3) spinal cord decompression due to disc extrusion.

## 2. Materials and Methods

This study was based on a retrospective review of medical records of patients who underwent surgical decompression for thoracolumbar disc extrusion and postoperative physiotherapy at the Rehabilitation Unit of the ‘I Portoni Rossi’ Veterinary Hospital between 2018 and 2024. It was conducted in accordance with European Union Directive 2010/63/EU. The Animal Welfare Body of the University of Bologna provided a positive ethical and scientific opinion on the publication of the data, certifying that this study did not involve animal experimentation but rather clinical veterinary practice (prot.74239/2025). Treatment consent was obtained from all owners, and the acupuncture protocols were performed by a licensed veterinarian with specific training in veterinary acupuncture (A.M.).

### 2.1. Inclusion and Exclusion Criteria

The patients were divided into two groups: dogs who received a physiotherapy protocol including acupuncture treatment (Group A) and a control group of dogs who underwent only standard physiotherapy treatment (Group B).

Specific inclusion criteria were established for patient selection:

- Presence of myelopathy secondary to thoracolumbar disc extrusion (T3–L3), diagnosed by a certified neurologist;
- Diagnostic confirmation of disc extrusion through magnetic resonance imaging (MRI);
- Surgical decompression via hemilaminectomy or mini-hemilaminectomy;
- Application of a physiotherapy protocol in the postoperative period, with hospitalization at the center or day-hospital attendance from Monday to Friday;
- Non-ambulatory paraparetic patients with preservation of deep pain sensation;
- Completion of at least three acupuncture sessions (for Group A);
- Minimum physiotherapy duration of at least 10 days to assess gait recovery;
- Absence of comorbidities (e.g., orthopedic conditions, other neurological disorders, metabolic diseases) or any other condition likely to negatively affect functional recovery (e.g., severely compromised patients following traumatic events, patients who underwent surgical revision due to complications in the immediate postoperative period).

Exclusion criteria:

- Incomplete medical records or lack of documentation regarding the exact time of observed gait recovery.

### 2.2. Parameters of Interest

For all included patients, the following data were recorded: breed, age, sex, body weight (kg), affected intervertebral space, time from symptom onset to surgery, and time from surgery to the start of rehabilitation. The severity of neurological deficits was assessed using a grade assigned to each patient based on the modified Frankel scale [19] at the beginning of rehabilitation.

Additionally, other parameters were evaluated: the time (in days) from the beginning of rehabilitation to the recovery of autonomous ambulation or, in cases where ambulation was not regained, the number of days of rehabilitation until discharge; and for Group A, the number of acupuncture treatments performed until the patient regained the ability to ambulate (number of sessions). A patient was considered ambulatory when able to take at least three consecutive steps autonomously (with no assistance) [20].

Postoperative treatment primarily involved the use of anti-inflammatory (meloxicam 0.1 mg/kg) and analgesic (Tramadol 3 mg/kg) drugs, sometimes supplemented with medication for neuropathic pain management (gabapentin 10 mg/kg), with dosages and durations adjusted to the individual needs of each patient.

### 2.3. Rehabilitation Protocol

All patients referred to the hospital’s physiotherapy unit underwent a comprehensive physiatric examination. The physiotherapy regimen for all patients consisted of two daily sessions (morning and afternoon), 5 days a week. Between sessions, the patients were confined to a cage with a soft mattress and taken out three to four times a day for approximately 5 min to allow urination and defecation. The physiotherapy protocol included the following: manual techniques (muscle massages), passive exercises (passive range of motion, stretching, flexor reflex), and assisted active exercises (standing maintenance using a physio roll, assisted walking, proprioceptive board, and assisted walking on an underwater treadmill). The underwater treadmill activity was introduced at increasing intensity and was included in the protocol only after surgical sutures were removed. Dogs from both groups also received daily laser therapy using the pre-set ‘muscle contractures’ program (targeting pectoral muscles, triceps brachii, quadriceps femoris, biceps femoris, and pectineus) and/or ‘IVDD’ program in the perilesional area (emitting sequential pulsed waves at a frequency of 18 Hz, a wavelength of 808 nm, and energy density of approximately 4 J/cm^2^). The laser handpiece was placed in contact with the skin, laterally to the spine, applying slight pressure at a 45° angle to the body’s median plane. Laser treatment was performed using a MPHI Vet Orange MLS® 75W (ASA srl, Arcugnano-VI-Italy).

All patients also underwent daily neuromuscular electrical stimulation (NMES) with a pre-set program for strengthening the hind limbs.

For patients in Group A, acupuncture and/or EA were integrated into the regimen, performed twice a week for the first 2–3 weeks, and then once a week after clinical improvement was observed. The type of treatment, as well as the number and frequency of sessions, varied depending on factors such as the patient’s responsiveness, temperament, and progression of neurological symptoms. Each session lasted 15–20 min for dry acupuncture and 20–30 min for electroacupuncture.

The needles used for dry acupuncture varied in diameter (0.15, 0.16, 0.18, 0.20, 0.25 mm) and length (15 or 30 mm), depending on the animal’s size, nutritional status, and the region being treated.

The device used for EA was a direct current pulse generator equipped with oscillating circuits that produced two types of waves: square waves and peak waves. These waveforms do not cause tissue overheating. Frequencies (cycles per second) ranged from a minimum of 1 to a maximum of 100 Hz. In this study, low frequencies (<15 Hz) were used. The current delivered was relatively low, ranging from 10 to 80 mA.

The selection of acupuncture points was tailored to the individual patient, the location of the IVDD, the desired neuromodulatory effects on the nervous and locomotor systems, and the principles of Traditional Chinese Veterinary Medicine regarding the use of local and distal points. Selected sites included the following acupuncture points: GV-14, posterior BaiHui, myofascial trigger points, BaLiao, BL-54, BL-40, BL-60, KI-3, KI-1, GB-30, GB-34, ST-36, SP-6, LI-4, SI-3, LIV-3.

EA was applied at the BaFeng and/or BaLiao points.

### 2.4. Statistical Analysis

The collected data were submitted to statistical analysis, reporting mean ± standard deviation (SD), median, and 95% confidence interval. The distribution and equality of variance of variables (age, weight, days between symptom onset and surgery, days between surgery and the start of rehabilitation, degree of neurological dysfunction, days required to regain ambulation, days of rehabilitation after which patients were discharged non-ambulatory, and the number of acupuncture sessions) were assessed using the Shapiro–Wilk test and Levene test, respectively.

For variables with non-normal distribution (age, weight, days between symptom onset and surgery, and days between surgery and the start of rehabilitation), the Mann–Whitney test was applied to identify differences between groups. The variable ‘days required to regain ambulation’ (DEA), which followed a normal distribution, was analyzed using an independent-samples *t*-test. Additionally, the success rate (percentage of dogs regaining ambulation) between groups was calculated using Equation (1):

Success rate = (dogs with regained ambulatory function [Group A or Group B]/all dogs [Group A or Group B]) × 100(1)

To assess whether the weight of the dogs could act as a covariate (confounding factor) influencing the recovery of autonomous ambulation, analysis of covariance (ANCOVA) was applied. The linear relationship between the dependent variable (recovery of ambulation) and the covariate (weight) was further tested using Pearson’s correlation, and the homogeneity of regression slopes was verified by calculating the regression estimation coefficient (β) for weight in Groups A and B [21].

Furthermore, considering the recovery of ambulation as a time-to-event factor, a multivariable Cox proportional hazards model was applied to evaluate the hazard ratio between groups, with groups, weight, and age included as covariates. The equation for the Cox model is presented as Equation (2):

h(t, X) = h0(t) × e^(β1Group+β2Weight+β3Age)^ (2)

Finally, Spearman’s correlation was used to evaluate the relationship between quantitative variables across all groups and within groups separately.

The statistical significance level was set at *p* < 0.05. Statistical analyses were performed using R 4.3.2 (R Foundation for Statistical Computing; Vienna, Austria; https://www.R-project.org/, accessed on 29 May 2024).

## 3. Results

The retrospective analysis of medical records identified a total of 155 patients referred to the physiotherapy unit following thoracolumbar disc herniation (T3–L3). However, 114 clinical cases were excluded because they did not meet the inclusion criteria for incomplete medical records or lack of documentation regarding the exact time of observed gait recovery. Therefore, 41 patients were definitely included and divided into Group A (18 patients—physiotherapy protocol with acupuncture) and Group B (23 patients—physiotherapy only).

In Group A, the most represented breed was the dachshund (n = 8). Other breeds in this group included the French bulldog (n = 3), mixed breed (n = 2), Labrador retriever (n = 1), Shar-Pei (n = 1), Jack Russell terrier (n = 1), West Highland white terrier (n = 1), and miniature poodle (n = 1), with a predominance of chondrodystrophic breeds. There were 7 males and 11 females in this group. The mean age was 5.8 ± 2.9 years (median, 5 years), and the mean body weight was 12.3 ± 2.9 kg (median, 12.3 kg). The mean interval between symptom onset and surgery was 1.72 ± 2.08 days (median, 1 day). The mean interval between surgery and the start of rehabilitation was 3.28 ± 1.67 days (median, 3 days). The mean number of days required for patients to regain ambulatory ability was 15.06 ± 6.38 days (median, 13.5 days). The mean number of acupuncture sessions, performed until ambulation was achieved, was 4.56 ± 1.15 (median, 4.5).

In Group B, the most represented breed was also the dachshund (n = 12). Other breeds in this group included mixed breed (n = 4), Bolognese (n = 2), Lagotto Romagnolo (n = 1), Maltese (n = 1), Labrador retriever (n = 1), golden retriever (n = 1), and French bulldog (n = 1), with a predominance of chondrodystrophic breeds. There were 15 males and 8 females in this group. The mean age was 7.4 ± 3.6 years (median, 6 years), and the mean body weight was 12.3 ± 12.6 kg (median, 7.5 kg). The mean interval between symptom onset and surgery was 1.43 ± 1.97 days (median, 1 day). The mean interval between surgery and the start of rehabilitation was 4.96 ± 2.62 days (median, 5 days). Patients in this group required a mean of 18.1 ± 6.43 days (median, 18.5 days) to recover ambulation. Five patients in Group B were discharged in a non-ambulatory state after 11, 14, 79, 111, and 29 days of rehabilitation, respectively.

In all dogs included in this study, the affected intervertebral space was between T10 and L3.

Statistical analysis revealed no statistically significant differences between the two groups for the variables age (*p* = 0.2003), weight (*p* = 0.5369), and time from symptom onset to surgery (*p* = 0.6567). However, a statistically significant difference was found for the variable time from surgery to the start of rehabilitation (*p* = 0.029), which was shorter in Group A.

The success rate, defined as the return to ambulation, was 100% in Group A and 78.26% in Group B (Equation (1)). However, no statistically significant difference was detected (*p* = 0.125 and *p* = 0.161 in the ANCOVA and *t*-test, respectively) regarding the number of days required for ambulatory recovery between Group A (acupuncture) and Group B (physiotherapy only). The covariance analysis also indicated that the variable weight could act as a confounding factor, significantly influencing the patient’s return to ambulation (*p* = 0.037) (Table 1).

Spearman’s correlation analysis revealed statistically significant relationships between some of the quantitative variables considered in this study. Specifically, a positive correlation was identified between age and the number of acupuncture sessions in Group A. This suggests that older patients required a greater number of acupuncture treatments before regaining ambulation (Table 2).

**Figure 1:**
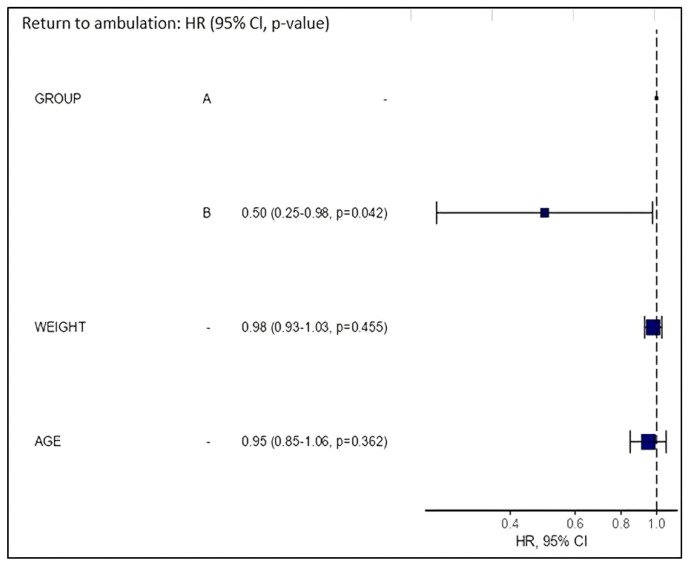
Hazard ratio graphs. Group B showed a hazard ratio of 0.50 and statistically significant differences in the return to ambulation compared with Group A (*p* = 0.042). Neither weight nor age had a statistically significant impact on the return to ambulation in this study (*p* = 0.455 and *p* = 0.362, respectively).

**Figure 2:**
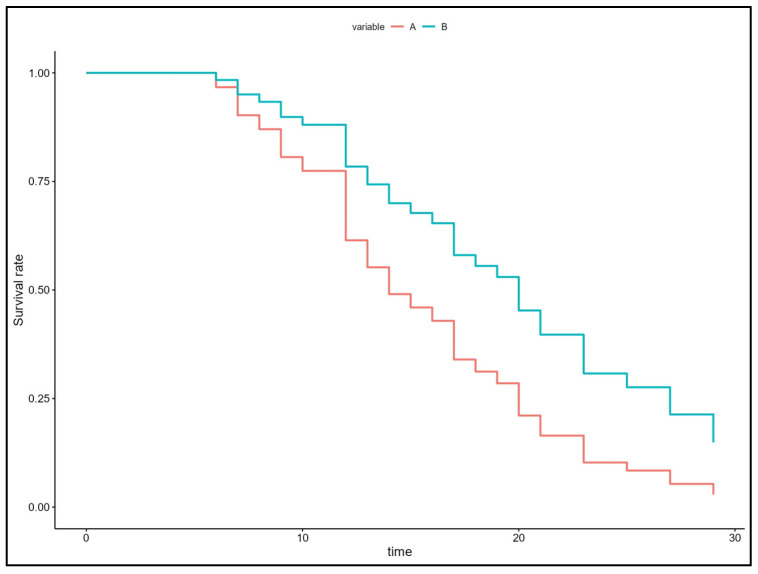
Curve of return to ambulation, according to the Cox model. The results indicate that patients in Group B had a 50% (hazard ratio = 0.50) lower probability of returning to ambulation over time compared with patients in Group A. Time is reported in the number of days.

The results of the Cox models and hazard ratio graphs are presented in Figure 1, while the curve for the return to ambulation is shown in Figure 2. These results indicate that patients in Group B had a 50% lower likelihood of regaining ambulation (hazard ratio = 0.50) over time compared with patients in Group A (*p* = 0.042).

## 4. Discussion

The aim of this study was to evaluate the positive effect of integrating acupuncture and EA treatments into a standard physiotherapy protocol for dogs undergoing surgery for thoracolumbar (T3–L3) disc extrusion. The likelihood of regaining ambulation was higher in the group that received acupuncture as part of the rehabilitation protocol. However, it should be noted that the dogs in Group B showed a slight delay (median of 5 days compared with 3 days in Group A) in starting physiotherapy after surgery.

In both groups, the breeds were of the chondrodystrophic type and had a median age of ≤6 years, consistent with the literature regarding the etiopathogenesis of intervertebral disc extrusion. The time between symptom onset and surgery is a key prognostic factor that can influence both the likelihood and speed of postoperative recovery [10,22]. In our study, all patients underwent decompressive surgery within 48 h of symptom exacerbation and subsequently received pain management therapy consistent with the literature (approximately 5–7 days) [10]. The 48-h window between the loss of deep pain perception (an event observed in two Group A dogs, two Group B dogs who regained ambulation, and two Group B dogs who did not) and decompressive surgery is considered optimal for the recovery of ambulation [10]. However, this time limit should not be interpreted as an absolute cut-off because the literature documents cases of plegic dogs lacking deep pain perception for >1 week prior to surgery regaining ambulation [10].

The selection criteria for our study excluded paraplegic dogs with a lack of deep pain perception at the start of rehabilitation. This decision was made to align with the most recent study by Jia et al. [23], which investigated the effects of acupuncture on patients with a similar condition. As previously reported, patients without deep nociception generally have a poorer prognosis for regaining ambulation, with a success rate of ≤60% [10,24,25,26], compared to patients with preserved deep pain perception. The latter group generally has a good to excellent prognosis (success rate > 90%) [10,26,27,28], with an average time to recovery of voluntary ambulation within 10–13 days [10,22,24,29,30,31]. Only a few publications have reported a slightly longer recovery time of around 16 days [32,33].

In our study, 100% of the dogs in Group A regained ambulation with a mean of 15.06 ± 6.38 days and a median of 13.5 days. By contrast, only 78.26% of the dogs in Group B regained the ability to walk independently, with a mean of 18.1 ± 6.43 days and a median of 18.5 days.

Despite the statistically significant difference in the interval between surgery and the start of rehabilitation (longer in Group B), all patients in this study began rehabilitation within the time frame recommended by scientific guidelines (24 h to 14 days postoperatively) [10].

The physiotherapy protocol design, in both groups, remained consistent in terms of the types of exercises and the progression of activities, while accounting for individual differences in the pace of neurological recovery. Regarding acupuncture treatment, the selected acupoints were the most commonly described for treating intervertebral disc herniation in dogs [14,16,34,35,36]. The choice of acupoints can depend on several factors, including the temperament of the patient and the type of lesion. In this study, the physiotherapist prioritized selecting a pool of acupoints capable of exerting synergistic effects with similar neuromodulatory impacts. Not all dogs tolerate needles equally, and not all dogs, even with lesions at the same thoracolumbar location between T3 and L3, present the same clinical symptoms. In particular, EA applied to the BaLiao points, although not commonly used in patients with T3–L3 disc herniations, is generally utilized in clinical cases requiring specific stimulation for micturition (neurogenic bladder) or in dogs exhibiting ‘lower motor neuron’ symptoms without actual involvement of the caudal spinal cord intumescence (e.g., due to spinal hemorrhage, leakage of extruded disc material, and similar conditions).

Moreover, the use of low frequencies for EA, as reported in previous studies [16,35], was selected for its potential benefits: generalized and long-lasting analgesia (through the release of opioid substances such as endorphins and enkephalins), better inhibition of neuropathic and inflammatory pain, and promotion of neuroplasticity [37,38]. It is also important to note that high frequencies are poorly tolerated by awake patients [1]. Nevertheless, other authors preferred alternating between low and high frequencies [14], suggesting that such a combination achieves the maximum therapeutic effect by releasing all four opioid peptides (dynorphin A, β-endorphin, endomorphin, and enkephalins), in line with Han’s study [16].

The outcomes and recovery times of ambulation in patients treated with acupuncture were found to be either better or in line with previous studies on the use of acupuncture and EA in treating dogs with intervertebral disc herniation [14,16,23,35,36]. These results also align with other clinical studies that employed more or less intensive rehabilitation protocols, excluding acupuncture, following surgical decompression [28,31,39].

Surgical intervention is highly recommended for non-ambulatory paraparetic or paraplegic dogs with deep pain perception because it is associated with significantly better success rates, recovery times, and lower recurrence probability compared with conservative approaches [10]. However, there are specific cases in which surgery may not be feasible (e.g., financial constraints or physical conditions that preclude anesthesia and surgery). In such cases, the integration of acupuncture and/or EA into the medical management plan can be a viable therapeutic option.

In clinical studies focusing on rehabilitation protocols after surgical decompression without acupuncture, Bruno et al. [28] reported recovery times comparable to those in Group A (acupuncture) in their study using a similar physiotherapy protocol. More recently, a retrospective clinical study by Martins et al. [39] analyzed clinical data from 367 paraplegic dogs with thoracolumbar disc extrusion. Their study demonstrated that, following decompressive surgery, an ‘intensive’ neurorehabilitation protocol (comprising massages, passive movements, locomotion training exercises, functional electrical stimulation, neuromuscular and interferential stimulation, and transcutaneous spinal cord electrical stimulation) effectively promoted ‘activity-induced neuroplasticity’, enabling faster recovery of ambulation compared with a standard physiotherapy protocol or spontaneous recovery [39]. At the same level of neurological dysfunction (paralysis with preserved nociception), the patients in their study also presented conditions such as absence or reduction of the flexor reflex, which contributed to a more challenging recovery [39]. Nonetheless, the success rate for patients with preserved nociception who underwent this type of intensive treatment was 99.4% (167/168), similar to the success rates observed in the present study.

In cases of acute disc extrusion, it can be challenging to attribute recovery to spontaneous neuroplasticity mechanisms rather than the rehabilitation protocol implemented. This consideration is less significant for patients who have lost deep pain perception, given their limited ability to recover autonomously. Therefore, it is plausible that the application of an intensive protocol in the postoperative management of patients with intervertebral disc herniation could be more beneficial for those with more severe neurological conditions [31].

In our study, patients with more severe neurological dysfunction were more prevalent in Group B, although the median modified Frankel scale score was not statistically different between the two groups. This could explain the seemingly faster recovery time, although not statistically significant, observed for the return to ambulation in Group A (acupuncture). In this study, acupuncture treatment does not appear to have significantly impacted the time to regain ambulation, but rather the actual likelihood of achieving this outcome, as indicated by the multivariate Cox proportional hazard model.

The decision to use a multivariate Cox proportional hazard model instead of a Kaplan–Meier curve was made to evaluate the potential influence of the variables weight and age on rehabilitation outcomes, in line with the literature [23,28,30,40,41,42]. The variables weight and age did not have a statistically significant effect on the event ‘return to ambulation’. However, it should be noted that among the non-ambulatory patients in Group B (n = 5), one was aged between 6 and 10 years, and two were aged >10 years, including one from a heavy breed (Labrador retriever). Two of the five patients did not regain ambulation after approximately 2 weeks of treatments (five physiotherapy sessions per week). Unfortunately, no useful information was available post-discharge, so it cannot be ruled out that ambulation might have been regained later, albeit over a particularly extended timeframe.

Although the results suggest a positive therapeutic effect of acupuncture treatment on neurological recovery, this study was limited by several technical aspects: a small sample size for statistical analysis, although consistent with several studies in the literature; the absence of a control group in which no patient received a rehabilitative treatment after surgery; or the absence of follow-up for non-ambulatory discharged patients; moreover, the retrospective nature of this study resulted in a lack of randomization, assessment, and monitoring of muscle atrophy and proprioception in all included patients, uniformity between the two groups on the timing of rehabilitation initiation, and validated pain scale monitoring.

## 5. Conclusions

The rehabilitative management of patients affected by thoracolumbar disc herniation undergoing decompressive surgery is a clear example of a multimodal approach. This approach involves the adoption of various strategies (manual therapies, instrumental therapies, and acupuncture) to address the main needs of the patient in the postoperative period: pain management, prevention of muscle atrophy and joint disorders secondary to the immobilization, stimulation, and recovery of proprioceptive function, and the stimulation of interneuronal circuits and neural mechanisms responsible for locomotor control, aiming to promote neuroplasticity.

The present study provides clinical insights supporting the inclusion of acupuncture and EA in a rehabilitation protocol, as treated dogs with acupuncture have a higher likelihood of regaining autonomous ambulation. However, further investigations are needed with randomized prospective studies.

## Figures and Tables

**Table 1:** Schematic results of Groups A and B.

	Age in Years	Weight (kg)	S-C (Days)	C-R (Days)	DEA (Days)
Group A	5.8 ± 2.9 (5) (4.31–7.24)	12.3 ± 2.9 (12.3) (7.38–13.4)	1.72 ± 2.08 (1) (0.69–2.76)	3.28 ± 1.67 (3) ^a^ (2.45–4.11)	15.06 ± 6.38 (13.5) (11.9–18.2)
Group B	7.4 ± 3.6 (6) (5.83–8.95)	12.3 ± 12.6 (7.5) (7.06–14.7)	1.43 ± 1.97 (1) (0.58–2.29)	4.96 ± 2.62 (5) ^a^ (3.82–6.09)	18.1 ± 6.43 (18.5) (14.9–21.3)

Schematic representation of the mean ± standard deviation (median) and 95% confidence interval of the values concerning the variables investigated in Groups A and B. S-C = time interval between the onset of symptoms and surgery; C-R = time interval between surgery and the start of rehabilitation; DEA = days from the start of rehabilitation until the patient regained ambulation; **^a^** = statistically significant difference (*p* < 0.05).

**Table 2:** Spearman correlation matrix—Group A.

GROUP	TEST	MATRIX	Age	Weight	S-C	C-R	T0	DEA	N. Session
A	Spearman correlation *p*-value	Age		0.1357	0.2084	0.7196	0.8362	0.5646	0.0086 *
		Weight	0.1357		0.6725	0.3176	0.8366	0.5212	0.6521
		S-C	0.2084	0.6725		0.1272	0.3698	0.5917	0.3027
		C-R	0.7196	0.3176	0.1272		0.0184	0.4823	0.6063
		T0	0.8362	0.8366	0.3698	0.0184		0.7216	0.8096
		DEA	0.5646	0.5212	0.5917	0.4823	0.7216		0.0012
		N. session	0.0086 *	0.6521	0.3027	0.6063	0.8096	0.0012	

S-C = time interval between the onset of symptoms and surgery; C-R = time interval between surgery and the start of rehabilitation; T0 = severity of neurological deficits according to the modified Frankel scale at the start of rehabilitation; DEA = time (expressed in days) from T0 required for the animal to regain ambulation; N. Sessions = number of acupuncture treatments performed until the return to ambulation. * indicates significant correlations.

## Data Availability

All data are contained in the manuscript.

## References

[1] Lindley S., Cummings M. (2006). Electroacupuncture and related techniques. Essentials of Western Veterinary Acupuncture.

[2] Harrison T.M., Churgin S.M. (2022). Acupuncture and traditional Chinese veterinary medicine in zoological and exotic animal medicine: A review and introduction of methods. Vet. Sci..

[3] Eul-Matern C. (2022). Acupuncture for Dogs and Cats: A Pocket Atlas.

[4] Huntingford J.L., Petty M.C. (2022). Evidence-Based Application of Acupuncture for Pain Management in Companion Animal Medicine. Vet. Sci..

[5] Wright B.D. (2019). Acupuncture for the Treatment of Animal Pain. Vet. Clin. N. Am. Small Anim. Pract..

[6] Fry L.M., Neary S.M., Sharrock J., Rychel J.K. (2014). Acupuncture for analgesia in veterinary medicine. Top. Companion Anim. Med..

[7] Habacher G., Pittler M.H., Ernst E. (2006). Effectiveness of acupuncture in veterinary medicine: Systematic review. J. Vet. Intern. Med..

[8] Sumida J.M., Matera J.M., Hayashi A.M. (2023). Randomized single-blinded prospective comparison between ozone therapy and electroacupuncture for canine thoracolumbar disk disease. Res. Vet. Sci..

[9] Bernardini M. (2010). Neurologia Del Cane e Del Gatto.

[10] Olby N.J., Moore S.A., Brisson B., Fenn J., Flegel T., Kortz G., Lewis M., Tipold A. (2022). ACVIM consensus statement on diagnosis and management of acute canine thoracolumbar intervertebral disc extrusion. J. Vet. Intern. Med..

[11] Lewis M.J., Granger N., Jeffery N.D., Canine Spinal Cord Injury Consortium (CANSORT-SCI) (2020). Emerging and adjunctive therapies for spinal cord injury following acute canine intervertebral disc herniation. Front. Vet. Sci..

[12] Spinella G., Bettella P., Riccio B., Okonji S. (2022). Overview of the current literature on the most common neurological diseases in dogs with a particular focus on rehabilitation. Vet. Sci..

[13] Frank L.R., Roynard P.F.P. (2018). Veterinary Neurologic Rehabilitation: The Rationale for a Comprehensive Approach. Top. Companion Anim. Med..

[14] Hayashi A.M., Matera J.M., De Campos Fonseca Pinto A.C.B. (2007). Evaluation of electroacupuncture treatment for thoracolumbar intervertebral disk disease in dogs. J. Am. Vet. Med. Assoc..

[15] Choi D.C., Lee J.Y., Moon Y.J., Kim S.W., Oh T.H., Yune T.Y. (2010). Acupuncture-mediated inhibition of inflammation facilitates significant functional recovery after spinal cord injury. Neurobiol. Dis..

[16] Han H.-J., Yoon H.-Y., Kim J.-Y., Jang H.-Y., Lee B., Choi S.H., Jeong S.-W. (2010). Clinical effect of additional electroacupuncture on thoracolumbar intervertebral disc herniation in 80 paraplegic dogs. Am. J. Chin. Med..

[17] Roynard P., Frank L., Xie H., Fowler M. (2018). Acupuncture for small animal neurologic disorders. Vet. Clin. N. Am. Small Anim. Pract..

[18] Santos B.P., Joaquim J.G., Cassu R.N., Pantoja J.C., Luna S.P.L. (2022). Effects of Acupuncture in the Treatment of Dogs with Neurological Sequels of Distemper Virus. J. Acupunct. Meridian Stud..

[19] Martin S., Liebel F.X., Fadda A., Lazzerini K., Harcourt-Brown T. (2020). Same-day surgery may reduce the risk of losing pain perception in dogs with thoracolumbar disc extrusion. J. Small Anim. Pract..

[20] Bennaim M., Porato M., Jarleton A., Hamon M., Carroll J.D., Gommeren K., Balligand M. (2017). Preliminary evaluation of the effects of photobiomodulation therapy and physical rehabilitation on early postoperative recovery of dogs undergoing hemilaminectomy for treatment of thoracolumbar intervertebral disk disease. Am. J. Vet. Res..

[21] Khammar A., Yarahmadi M., Madadizadeh F. (2020). What is analysis of covariance (ancova) and how to correctly report its results in medical research?. Iran. J. Public Health.

[22] Ferreira A., Correia J., Jaggy A. (2002). Thoracolumbar disc disease in 71 paraplegic dogs: Influence of rate of onset and duration of clinical signs on treatment results. J. Small Anim. Pract..

[23] Jia Q., Wang Y., Pang H., Fan K., Xie H., Lin J. (2023). Retrospective study of acupuncture treatment for canine thoracolumbar intervertebral disc herniation. One Health Adv..

[24] Aikawa T., Fujita H., Kanazono S., Shibata M., Yoshigae Y. (2012). Long-term neurologic outcome of hemilaminectomy and disk fenestration for treatment of dogs with thoracolumbar intervertebral disk herniation: 831 cases (2000–2007). J. Am. Vet. Med. Assoc..

[25] Jeffery N.D., Barker A.K., Hu H.Z., Alcott C.J., Kraus K.H., Scanlin E.M., Granger N., Levine J.M. (2016). Factors associated with recovery from paraplegia in dogs with loss of pain perception in the pelvic limbs following intervertebral disk herniation. J. Am. Vet. Med. Assoc..

[26] Langerhuus L., Miles J. (2017). Proportion recovery and times to ambulation for non-ambulatory dogs with thoracolumbar disc extrusions treated with hemilaminectomy or conservative treatment: A systematic review and meta-analysis of case-series studies. Vet. J..

[27] Shaw T.A., De Risio L., Laws E.J., Rose J.H., Harcourt-Brown T.R., Granger N. (2017). Prognostic factors associated with recovery of ambulation and urinary continence in dogs with acute lumbosacral spinal cord injury. J. Vet. Intern. Med..

[28] Bruno E., Canal S., Antonucci M., Bernardini M., Balducci F., Musella V., Mussoni M., Spinella G. (2020). Perilesional photobiomodulation therapy and physical rehabilitation in post-operative recovery of dogs surgically treated for thoracolumbar disk extrusion. BMC Vet. Res..

[29] Davis G.J., Brown D.C. (2002). Prognostic indicators for time to ambulation after surgical decompression in nonambulatory dogs with acute thoracolumbar disk extrusions: 112 cases. Vet. Surg..

[30] Ruddle T.L., Allen D.A., Schertel E.R., Barnhart M.D., Wilson E.R., Lineberger J.A., Klocke N.W., Lehenbauer T.W. (2006). Outcome and prognostic factors in nonambulatory Hansen Type I intervertebral disc extrusions: 308 cases. Vet. Comp. Orthop. Traumatol..

[31] Zidan N., Sims C., Fenn J., Williams K., Griffith E., Early P.J., Mariani C.L., Munana K.R., Guevar J., Olby N.J. (2018). A randomized, blinded, prospective clinical trial of postoperative rehabilitation in dogs after surgical decompression of acute thoracolumbar intervertebral disc herniation. J. Vet. Intern. Med..

[32] Hady L.L., Schwarz P.D. (2015). Recovery times for dogs undergoing thoracolumbar hemilaminectomy with fenestration and physical rehabilitation: A review of 113 cases. J. Vet. Med. Anim. Health.

[33] Hodgson M., Bevan J., Evans R., Johnson T. (2017). Influence of in-house rehabilitation on the postoperative outcome of dogs with intervertebral disk herniation. Vet. Surg..

[34] Yang J.W., Jeong S.M., Seo K.M., Nam T.C. (2003). Effects of corticosteroid and electroacupuncture on experimental spinal cord injury in dogs. J. Vet. Sci..

[35] Joaquim J.G.F., Luna S.P.L., Brondani J.T., Torelli S.R., Rahal S.C., De Paula Freitas F. (2010). Comparison of decompressive surgery, electroacupuncture, and decompressive surgery followed by electroacupuncture for the treatment of dogs with intervertebral disk disease with long-standing severe neurologic deficits. J. Am. Vet. Med. Assoc..

[36] Liu C.M., Lin C.T. (2015). Retrospective Study of a New Standardized Acupuncture Treatment Protocol on Thoracolumbar Spinal Cord Diseases in 84 Dogs. Pak. Vet. J..

[37] Dewey C., Xie H. (2021). The scientific basis of acupuncture for veterinary pain management: A review based on relevant literature from the last two decades. Open Vet. J..

[38] Zhang R., Lao L., Ren K., Berman B.M. (2014). Mechanisms of acupuncture–electroacupuncture on persistent pain. Anesthesiology.

[39] Martins Â., Gouveia D., Cardoso A., Carvalho C., Coelho T., Silva C., Viegas I., Gamboa Ó., Ferreira A. (2021). A controlled clinical study of intensive neurorehabilitation in post-surgical dogs with severe acute intervertebral disc extrusion. Animals.

[40] Brisson B.A. (2010). Intervertebral disc disease in dogs. Vet. Clin. N. Am. Small Anim. Pract..

[41] Draper W.E., Schubert T.A., Clemmons R.M., Miles S.A. (2012). Low-level laser therapy reduces time to ambulation in dogs after hemilaminectomy: A preliminary study. J. Small Anim. Pract..

[42] Olby N., Levine J., Harris T., Muñana K., Skeen T., Sharp N. (2003). Long-term functional outcome of dogs with severe injuries of the thoracolumbar spinal cord: 87 cases (1996–2001). J. Am. Vet. Med. Assoc..

